# The combination of NPK fertilizer and deltamethrin insecticide favors the proliferation of pyrethroid-resistant *Anopheles gambiae* (Diptera: Culicidae)

**DOI:** 10.1051/parasite/2012192159

**Published:** 2012-05-15

**Authors:** F. Darriet, M. Rossignol, F. Chandre

**Affiliations:** 1 Institut de Recherche pour le Développement (IRD), Unité Mixte de Recherche (UMR) Maladies Infectieuses et Vecteurs, Écologie, Génétique, Évolution et Contrôle (MIVEGEC), Laboratoire de Lutte contre les Insectes Nuisibles (LIN) 911, avenue Agropolis BP 64501 34394 Montpellier Cedex 5 France

**Keywords:** NPK fertilizer, plant matter, deltamethrin, pyrethroidresistance, proliferation, *Anopheles gambiae*, engrais NPK, matière végétale, deltaméthrine, résistance, pyréthrinoïdes, prolifération, *Anopheles gambiae*

## Abstract

In this laboratory study, we investigated how the biological cycle of *Anopheles gambiae* s.s. (VKPR strain) would be like when grew in an environment containing more or less plant matter (2.5 or 5 g/l) and fertilizer (8-12-8 or 17-23-17 mg/l). Half of the environments studied were not exposed to insecticide (control) whereas the other half was submitted to deltamethrin treatment at the concentration of 0.015 mg/l. The bioassays showed that 2.5 g/l of plant matter in water are not sufficient to feed the hundred larvae, each breeding site contains. Treating these breeding sites with deltamethrin reversed the situation as it decreased the competition for food resources and allowed the surviving larvae to share the small amount of food enabling them to pursue their development until adults. If the introduction of NPK in untreated sites has not improved the nutritive qualities of the water, in the treated sites it multiplied the number of emerging adults by 2.5. In the waters containing 5 g/l of plant matter, the larvae did not undergo feeding competition and the impact of insecticide followed of a more traditional selection scheme that expressed itself by a lower number of emerging adults. In these environments treated or nontreated where plant matter is abundant, adding NPK brings food supplement to the larvae therefore increases the survival rate of *An. gambiae*. To conclude, whether in habitats with little or much plant matter, NPK presence in water results in larger adults with generally, more soluble proteins.

## Abstract (fr)

Dans cette étude de laboratoire, les auteurs ont étudié le cycle biologique d*’Anopheles gambiae* s.s. résistant aux pyréthrinoïdes (souche VKPR) selon que celui-ci se développe dans un environnement plus ou moins riche en matière végétale (MV = 2,5 et 5 g/l) et en engrais NPK (8-12-8 et 17-23-17 mg/l). La moitié des environnements composés n’a subi aucune pression de sélection (gîtes témoins) alors que l’autre moitié a fait l’objet d’un traitement à la deltaméthrine à la concentration de 0,015 mg/l. Les essais ont montré que 2,5 g de MV par litre d’eau ne suffisent pas à nourrir la centaine de larves contenue dans chaque gîte. Le fait de traiter ces gîtes avec la deltaméthrine inverse toutefois la situation en donnant aux larves survivantes l’occasion de se partager le peu de nourriture disponible et de poursuivre ainsi leur développement jusqu’au stade adulte. Si l’apport en NPK dans les gîtes non traités n’a pas amélioré les qualités nutritives des eaux, cet apport dans les gîtes traités a multiplié par 2,5 le nombre des adultes émergents. Dans les eaux contenant 5 g de MV par litre, les larves n’ont pas subi de stress alimentaire et l’impact de l’insecticide a suivi un schéma de sélection plus classique qui s’est exprimé par une réduction du nombre des adultes émergents. Dans ces environnements (traités ou non) où abonde la matière organique, l’ajout de NPK apporte aux larves un complément nutritionnel qui augmente plus encore le taux de survie d*’An. gambiae*. Enfin, que ce soit dans les milieux pauvres ou riches en MV, la présence de NPK dans l’eau engendre des adultes plus gros et généralement plus riches en protéines solubles.

## Introduction

Among all the anopheles transmitting malaria to man, *Anopheles gambiae* s.l. Giles is by the most efficient vector in the world. According to the World Health Organization estimations (WHO, 2009), two billion people live in areas plagued by endemic malaria. This disease is responsible for three hundred to five hundred million clinical cases each year, more than 90 % of which occurring on the African continent. *An. gambiae* larvae proliferate in clear water collections exposed to the sun, such as ponds, puddles and rice fields. Moreover the densities of anopheles larvae in rice paddies have been reported reaching peaks a few days after the pouring of nitrogenous fertilizer at the time of transplanting (Victor & Reuben, 2000; Mwangangi *et al.*, 2006). For other mosquitoes like, the dengue vector *Aedes aegypti*, demonstration was made in laboratory that the presence of NPK fertilizer in water attracted the gravid females looking for a place to lay their eggs. Also, an NPK fertilizer combined to plant matter favored the development of larvae, hence the emergence of adult mosquitoes (Darriet & Corbel, 2008a; Darriet *et al.*, 2010a).

Currently, *An. gambiae* has become resistant to common classes of insecticides throughout Africa (Chandre *et al.*, 1999; N’guessan *et al*., 2003; Darriet, 2007; Ranson *et al.*, 2011) thus representing a threat to the efficacy of vector control programs based on pyrethroids for indoor residual spraying or long-lasting nets. The widespread resistance of *An. gambiae* to pyrethroids partly results from the massive use of these insecticides in agriculture (Diabate *et al.*, 2002; Yadouleton *et al.*, 2011). Soils easily absorb the pyrethroids (Harris *et al.*, 1981) which rain water drains into the rice fields and other water collections. The *An. gambiae* larvae that grow in such an environment are inevitably exposed to intense and long-lasting selection pressures that affect their susceptibility to insecticides.

Taking rice paddies as a model with their abundant *An. gambiae* mosquito populations, we attempt to find out how the biological cycles of resistant and susceptible strains of *An. gambiae* s.s. would be like when grew in an environment containing more or less plant matter and fertilizer, and how they are modified when larvae are exposed to an insecticide treatment, here deltamethrin. *An. gambiae* females from untreated environments were submitted to susceptibility tests using deltamethrin while those emerging from treated environments were weighed and analyzed to determine their soluble protein content.

## Materials and Methods

### Mosquito strains

Two strains of *An. gambiae* s.s. were used in this study. A susceptible strain KIS (S form) originating from Kisumu in Kenya and a strain resistant to pyrethroids (VKPR: homozygous for knockdown resistance (*Kdr*) Leu-1014-Phe) originating from Kou Valley in Burkina-Faso have been maintained for 15 years under laboratory conditions (27 ± 2 °C and 80 % RH). The VKPR strain (M form) was already resistant to pyrethroid on the field and has been maintained subsequently under constant permethrin selection until homozygosis for *Kdr* (Darriet *et al.*, 1997).

### NPK fertilizer, plant matter and insecticide

The NPK liquid fertilizer 5-7-5 (Algoflash^®^) contained 5 % of nitrogen (N) consisting in 3 % of nitric nitrogen (NO_3_^-^) and 2 % of ammoniacal nitrogen (NH_4_^+^), 7 % of phosphorus (P) in the form of phosphorus pentoxide (P_2_O_5_), 5% of potassium (K) in the form of potassium oxide (K_2_O). The plant matter (PM) composed of dry grasses (hay) is sold in pet shop for the feeding of rabbits and little rodents (Zolux^®^). The insecticide treatments were made by using 100 % technical grade of deltamethrin (Δ) (Hoechst and Schering, Berkhamsted, UK).

### Combination of PM-NPK fertilizer and Δ

**Fig. 1.**
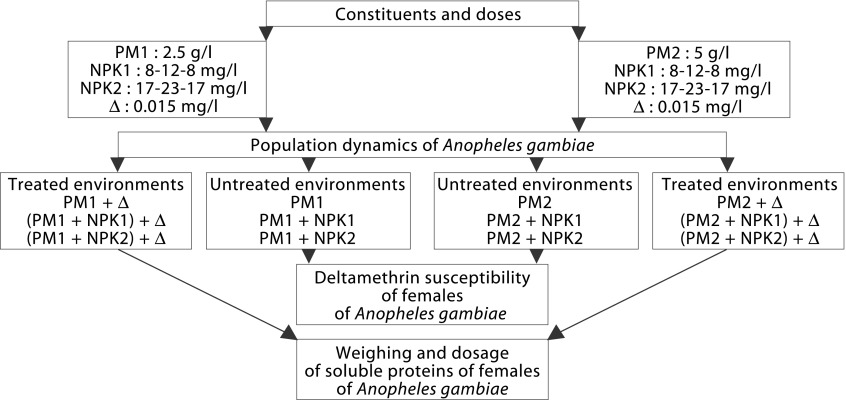
Quantities of plant matter (PM), NPK fertilizer and deltamethrin (Δ) included in the combination of the environments evaluated on *Anopheles gambiae* VKPR and KIS strains. *An. gambiae* females from untreated environments were submitted to susceptibility tests using deltamethrin while those emerging from treated environments were weighed and analyzed to determine their soluble protein content.

From two different concentrations of PM (2.5 and 5 g/ l) and NPK (8-12-8 and 17-23-17 mg/l) and the dose of 2.5 g/ha of Δ, 12 environments (Fig. 1) were prepared in 0.06 m^2^ plastic trays, each containing one liter of osmosed water. The two NPK doses chosen had already been studied on *Ae. aegypti* mosquito (Darriet & Corbel, 2008a; Darriet et al., 2010a). The 2.5 g/ha (= 0.25 mg/m^2^) deltamethrin dose is the one currently applied in rice fields to control rice gall midge *Orseolia oryzivora* (Diptera: Cecidomyiidae) (Dakotto & Nacro, 1987). In our one liter plastic trays the deltamethrin concentration is equivalent to 0.015 mg/l. The artificial environments were all evaluated on a total of three replicates, except for PM1, PM2, PM1 + Δ et PM2 + Δ that were done on six replicates.

### Population dynamics of adults of *An. Gambiae* VKPR and KIS strains in accordance with the environments

24 hr after the preparation of artificial environments, 100 1st instars larvae (L1) of *An. gambiae* were numbered and placed in each tray, their food resources depending solely on the constitutive elements of environments (plant matter and/or NPK fertilizer). Throughout the duration of the experiment, the trays were maintained at temperature of 27 ± 2 °C in the laboratory. The treatments using deltamethrin were done 48 hr after larval introduction in tray. All 12 environments treated or not (Fig. 1) were evaluated with *An. gambiae* VKPR pyrethroid-resistant strain. With *An. gambiae* KIS strain susceptible to insecticides, the assays were carried out only in the treated environments. Male and female adults were counted daily to establish the population dynamic specific to each environment. The data that were collected were expressed in cumulative percentages emergence along time, for each point on the curve a confidence interval (95 %) was calculated according the Wald method (Vollset, 1993).

### Deltamethrin susceptibility of females of *An. Gambiae* VKPR and KIS strains

In order to measure the impact of NPK fertilizer on the susceptibility of *An. gambiae* VKPR strain, the females emerging from non-treated environments were exposed filter papers treated at the discriminating dosage of deltamethrin (0.05 % = 18,4 mg/m^2^). The reference data were obtained with *An. gambiae* KIS strain raised in the insectarium. Filter papers were treated using acetone solution of insecticide and silicone oil as the carrier (WHO, 2006). Mortality was recorded 24 hr after exposure, corrected by the formula of Abbott if necessary (Abbott, 1925). The mortality rates were compared using a χ^2^ test with Yates’ correction.

### Weight of the VKPR females and dosage of soluble proteins

The females from the treated breeding sites were frozen at - 40 °C and a sample of 35 females from each environment was weighed in groups of five on a precision scale and each of the females was individually put in eppendorf tubes to be crushed in 150 μl distilled water. After being centrifuged the homogenates at 7,500 rpm for 5 mn at 4 °C, 10 μl of each supernatant were dropped into two out of the 96 wells of a plaque. The standard range of the soluble proteins [50, 100, 250, 500, 750 and 1,000 μg/ml of bovine serum albumin; Bradford-protein assay kit (Oz Biosciences, Marseille, France)] were also put on the plaque following the same procedure. Once the supernatants and the protein standard range solutions were deposited in the wells, 140 μl of Coomassie brilliant blue were added (Bradford, 1976). The quantities of soluble proteins were measured with a spectrophotometer at 595 nm (Wallac Victor 2). The average weights of females and the quantities of soluble proteins were compared in pairs, depending on the origin of the environment, by means of a Student’s t-test for independent samples (Statistica, 2006).

## Results

### Population dynamics of adults of *An. Gambiae* VKPR strain in accordance with the environment

The population dynamics of VKPR strain were very different depending on the composition of the environment.

**Fig. 2.**
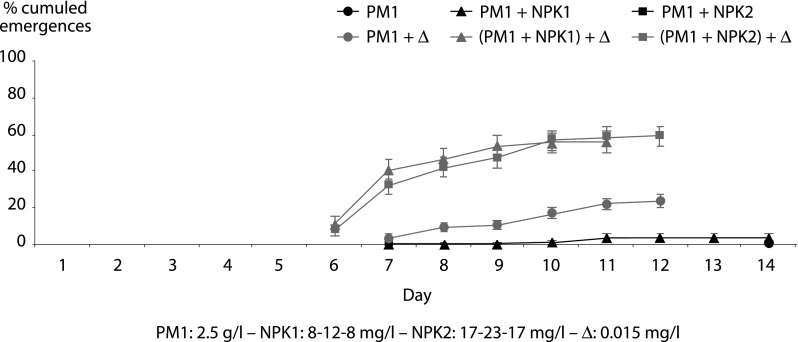
Cumulated percentages of male and female adults of *Anopheles gambiae* VKPR strain whose larvae lived in environments composed of plant matter (PM1) alone or in combination with a NPK fertilizer, treated or untreated with deltamethrin (Δ).

• PM1: 2.5 g/l – NPK1: 8-12-8 mg/l – NPK2: 17-23-17 mg/l – Δ: 0.015 mg/l (Fig. 2)

Less than 4 % of the adults were collected in untreated environments. The dose of 2.5 g/l of plant matter was not sufficient to provide enough food to the 100 larvae of *An. gambiae* VKPR and the addition of NPK in the water did not increase the food resources. When the environments were treated with deltamethrin, paradoxically more adults emerged than in untreated breeding sites with a cumulative emergence of 24 % at D12 in PM1 + Δ, 56 % in (PM1 + NPK1) + Δ and 59 % in (PM1 + NPK2) + Δ. Adding NPK1 or NPK2 concentrations to PM1 + Δ favored the larval growth and the adults emergence, without any significant difference between both fertilizer doses.

**Fig. 3.**
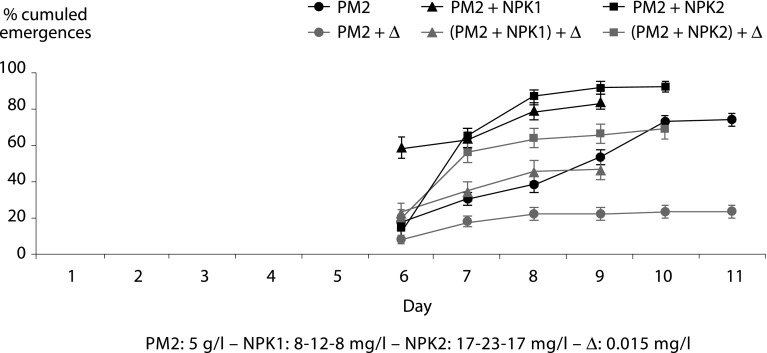
Cumulated percentages of male and female adults of *Anopheles gambiae* VKPR strain whose larvae lived in environments composed of plant matter (PM2) alone or in combination with a NPK fertilizer, treated or untreated with deltamethrin (Δ).

• PM2: 5 g/l – NPK1: 8-12-8 mg/l – NPK2: 17-23-17 mg/l – Δ: 0.015 mg/l (Fig. 3)

Doubling the dose of plant matter (PM2) allowed the larvae of *An. gambiae* VKPR to have a normal growth and after 8 to 11 days following the beginning of the test to induce 74 % emergence in PM2, 84 % in PM2 + NPK1 and 93 % in PM2 + NPK2. In these experiments, the treatment of breeding sites with deltamethrin reduced the percentage of adult emergence to 23 % in PM2 + Δ (similar to PM1 + Δ), 47 % in (PM2 + NPK1) + Δ and 69 % in (PM2 + NPK2) + Δ. A dose effect of NPK on the nutritive qualities was observed in both untreated and treated environments. Also it is worth noticing that (PM2 + NPK2) + Δ treated environment gave adult emergence rate almost as high as with untreated plant matter (PM2) alone (74 and 69 % respectively).

### The population dynamics of *An. gambiae* KIS strain in accordance with the environments

Deltamethrin at the concentration of 0.015 mg/l killed 100 % of the larvae of *An. gambiae* KIS susceptible to pyrethroids whatever the environment.

### Deltamethrin susceptibility of females of *An. gambiae* VKPR and KIS strains

The bioassays were carried out with adult females from untreated habitats. Due to the very small individuals collected in PM1 environments, the tests were only performed with the adults emerging from PM2, PM2 + NPK1 and PM2 + NPK2 environments (Table I). Compared to susceptible KIS, VKPR strain confirmed its high level of resistance to pyrethroids (0.0003 < *P* < 0.01). VKPR females from PM2 + NPK1 and PM2 + NPK2 demonstrated a susceptibility to deltamethrin similar to PM2 (respectively *P* = 0.98 and *P* = 0.27).

**Table I.** Activity of deltamethrin at a discriminating dosage (0.05 % = 18,4 mg/m^2^) against the pyrethroid-resistant strain of *Anopheles gambiae* (VKPR) and the insecticide-susceptible strain of *An. gambiae* (KIS).

		Number of females tested	Mortality 24 h (95% CI)
VKPR	PM2	84	91.7 (85.8–97.6)
	PM2 + NPK1	61	91.8 (85.0–98.6)
	PM2 + NPK2	72	86.1 (78.1–94.1)
KIS	Insectarium	105	100

**Table II.** Average weights and soluble protein contents in females of *Anopheles gambiae* VKPR strain.

Environment	Average weights (mg) per female (95% CI)	Average of soluble proteins (mg) per female (95% CI)
PM1 + Δ	0.77 (0.74–0.80)	0.051 (0.047–0.055)
(PM1 + NPK1) + Δ	1.12 (1.04–1.20)	0.089 (0.081–0.096)
(PM1 + NPK2) + Δ	0.97 (0.93–1.01)	0.066 (0.060–0.071)
PM2 + Δ	1.18 (1.11–1.25)	0.073 (0.068–0.079)
(PM2 + NPK1) + Δ	1.42 (1.34–1.49)	0.083 (0.076–0.090)
(PM2 + NPK2) + Δ	1.60 (1.50–1.70)	0.079 (0.073–0.086)

### Average weights and protein contents of female *An. gambiae* VKPR (Table II)

The average weight and the dosage of soluble proteins were measured on the females emerging from the environments treated with deltamethrin. Doubling the quantity of plant matter in the water (MV1: 2.5 g/l *versus* MV2: 5 g/l) led to the emergence of larger females, containing more soluble proteins (*P* < 0.0001). The females of (PM1 + NPK1) + Δ and (PM1 + NPK2) + Δ showed average weights and protein contents greater than in adults emerged from PM1 + Δ (*P* < 0.0001). The addition of NPK to an environment lacking organic matter is enough to raise larger mosquitoes with more proteins.

The females of (PM2 + NPK1) + Δ and (PM2 + NPK2) + Δ showed average weights greater than the females collected in PM2 + Δ (*P* < 0.001) while having barely significant more proteins (*P* = 0.027) for (PM2 + NPK1) + Δ and non-significant ones (*P* = 0.16) for (PM2 + NPK2) + Δ. Therefore the addition of NPK to an organic matter filled habitat favors the emergence of bigger females of *An. gambiae* not necessarily richer in soluble proteins.

## Discussion

Rice fields are environments favorable to the proliferation of *An. gambiae* and it is now well established that the levels of proliferation of this mosquito is in relation with the cultivating techniques adopted by the farming communities. With our laboratory study we attempted to identify some of the physico- chemical factors favoring the larval development of pyrethroid-resistant *An. gambiae* VKPR whether the breeding sites contained plant matter alone or in association with NPK fertilizer. Half of the environments studied were not exposed to selection pressure (control) whereas the other half was submitted to deltamethrin treatment.

The bioassays showed that 2.5 g/l plant matter were not enough to feed the hundred of larvae. Treating the breeding sites with deltamethrin (0.015 mg/l) reversed the situation as it decreased the competition for food resources and allowed the surviving larvae to share the small amount of food enabling them to pursue their development into adults. If adding NPK (8-12-8 mg/l and 17-23-17 mg/l) did not improve the nutritive qualities of untreated habitats, it significantly impacted the quality of treated ones by multiplying by 2.5 the number of emerging adults (23.5 % *versus* 56 and 59 %). The addition of NPK in a poor environment treated with deltamethrin also noticeably impacted the average weight of the females and their soluble protein contents.

On the other hand the environments containing 5 g/l of plant matter (PM2) provided enough food (proteins, glucids and lipids) for the larvae and allowed an emergence rate of 74 %. Both NPK concentrations brought nutritional supplement compared with plant matter alone and resulted in the emergence of a greater number of adults (74 % *versus* 84 and 93 %). Actually, nitrogen, phosphorus and potassium are not directly assimilated by the mosquito larvae. However the three minerals enhance the development of bacteria, algae and fungi, increasing the food biomass of the breeding sites. Insofar as MV2 environments alone or in combination with NPK did not generate feeding competition, the impact of treatment went along a more traditional selection scheme by decreasing the number of adult emergences. Also if the females from those sites were characterized by the greatest average weights of the study, quite paradoxically the protein contents of the females PM2 + NPK1 and PM2 + NPK2 mixtures were not more important than for those emerging in PM2 alone. In such environments, rich in organic matter, mosquito larvae produce the protein amount they need, the rest of the food being turned into lipids (fatty acids, phospholipids and sterols) that can constitutes up to 50 % of the insect body (Gilbert & Chino, 1974). In any case the gain of proteins and/or lipids in VKPR *An. gambiae* females does not show any significant incidence on the expression of resistance to deltamethrin.

The carbon (C) and potassium (K) concentrations in water where 5 g of plant matter were added were high whereas mineral nitrogen contents (NH_4_^+^ + NO_3_^-^) and phosphorus (P) remained low (Darriet *et al*., 2010a). A mixture made of plant matter and NPK fertilizer adds up the supplies each part brings into an organic-mineral complex rich in nutrients. Nitrogen is the most abundant atom next to carbon in the chemistry of the living. Among most insects, the fertility of females is often correlated with the amount of nitrogen in the ingested food (Scriber & Slansky, 1981; Ridsdill-Smith, 1991). Phosphorus is absorbed by the cells for the synthesis of the nucleic acids, and phospholipids whereas potassium is more specifically involved in the making of proteins (Magigan & Martinko, 2007). Mosquito larvae ingest large quantities of organic matter in their diet, which results in a reduction of turbidity in the water and allows organic nitrogen to proceed to mineralization *via* the nitrifying bacteria (Darriet & Corbel, 2008b).

In sites lacking organic matter and unable to ensure the feeding of a great number of mosquito larvae, the addition of NPK is not enough to compensate the nutritional deficit. Paradoxically, treating these unfavorable sites – by eliminating only part of the larvae of *An. gambiae* VKPR – allows the surviving ones to consume the food available and keep growing into adult stages. This selective pressure generated by the insecticide, making breeding sites formerly improper to mosquito development into a productive one, can be only observed in regions where *An. gambiae* is resistant to pyrethroids. Indeed, in our study we have observed that treating similar breeding sites at the same dosage of deltamethrin eliminated all the susceptible larvae from Kisumu strain. In the place where *An. gambiae* shows limited susceptibility to conventional insecticides (pyrethroids, organophosphorus and carbamates), it is now necessary to use, at least in rice fields, insecticides that are active on new targets, or combinations of insecticides with different modes of action, that can eliminate resistant mosquitoes (Darriet *et al*., 2010b; Darriet & Chandre 2011).

Finally this study shows us how the not so well thought-out use of pesticides and fertilizers in agriculture could impact the mosquito environment and even generates new ecological systems beneficial to the proliferation of mosquitoes. They cause a growing eutrophication of the natural breeding sites and select for resistance mechanisms to insecticides, already widespread among nuisance and disease vectors mosquitoes.
